# A Prospective Observational Study of Infantile Dermatoses in Infants Aged 29 Days to 12 Months at a Rural Tertiary Care Center in North India

**DOI:** 10.7759/cureus.117110

**Published:** 2026-09-28

**Authors:** Yuti Mishra, Paulina Chaudhary, Guramrit Singh, Mudita Gupta, Nidhi Yadav, Kaveri Arora, Gurdeep Dhanjal, Riya Goyal

**Affiliations:** 1 Dermatology, Adesh Medical College & Hospital, Ambala, IND; 2 Pediatrics, Adesh Medical College & Hospital, Ambala, IND

**Keywords:** atopic dermatitis, dermatoses, diaper dermatitis, infant, infective dermatoses, scabies

## Abstract

Background: The period of infancy is marked by a crucial physiological shift, during which the skin undergoes rapid development, leading to modifications in immune responses and physical protection. The skin of an infant represents a transitional stage - retaining certain physiological characteristics of neonatal skin while gradually acquiring the functional maturity of adult skin. This study aimed to diagnose and categorize cutaneous lesions in children between 29 days and 12 months of age and to identify specific dermatoses that coexisted with traditional rural child-rearing practices (e.g., oil massage and application of cow dung/kajal).

Methods: A prospective observational study was conducted among 200 infants aged 29 days to 12 months with cutaneous changes attending the dermatology outpatient department of a tertiary care teaching hospital in North India.

Results: The study included 200 infants (29 days to 12 months) comprising 115 males (57.5%) and 85 females (42.5%). Concerning age distribution, the largest proportion of infants fell in the 6-12 month age group (n = 90, 45%). Most of the infants belonged to a rural locality (n = 128, 64%) and lower-middle socioeconomic class (n = 110, 55%). Oil massage was practiced in the majority of infants (n = 160, 80%). Traditional cosmetic/remedial practices were also documented, with kajal/surma application being the most frequent (n = 64, 32%), followed by talcum powder use (n = 31, 15.5%). A wide spectrum of dermatological conditions was observed across the study population. Eczematous and inflammatory dermatoses emerged as the single largest category overall (n = 93, 46.5%). Atopic dermatitis was the leading diagnosis within this group (n = 28, 14%), closely followed by diaper dermatitis (n = 25, 12.5%), followed by seborrheic dermatitis/cradle cap (n = 22, 11%), and irritant contact dermatitis (n = 15, 7.5%). Bacterial infections were the next most prevalent group, followed by fungal infections, parasitic infestation, viral infections, congenital and developmental lesions, and lastly pigmentary disorders.

Conclusion: Most studies on infantile dermatoses originate from high-volume referral centers and predominantly focus on the neonatal period. Limited data are available from lower-volume tertiary care centers serving predominantly rural populations. Our study specifically evaluated infants aged 29 days to 12 months, focusing on acquired infectious and inflammatory dermatoses and excluding the neonatal period. This provides insight into the disease spectrum, preventable morbidity, and potentially modifiable household practices in this relatively understudied age group.

## Introduction

Infancy is a dynamic period of physiological development during which the skin undergoes significant structural and functional maturation. Although infant skin retains several features of neonatal skin, it gradually acquires the characteristics and functional maturity of adult skin. These developmental changes may influence the susceptibility and clinical presentation of various dermatoses during infancy. The epidermal barrier, though more competent than at birth, remains thinner and more permeable than adult skin, making infants susceptible to irritants, infections, and trans-epidermal water loss [1]. At the same time, increasing sebaceous activity, maturation of immune responses, and an evolving skin microbiome move the infant closer to the adult cutaneous profile. The skin barrier and immune systems are immature, so dermatological problems are common and can progress rapidly from benign to serious. The spectrum of dermatosis is different from other age groups. This ambiguity is more pronounced in the rural population, which may lead to detrimental outcomes either due to potentially harmful traditional remedies by anxious parents or significant pathological markers being neglected because of a lack of specialized awareness. The skin of an infant is not merely a smaller version of adult skin; it is a complex organ in a state of functional and structural maturation. Infant skin, although more mature than neonatal skin, does not yet possess the full structural integrity and immunological competence of adult skin. It exists in a transitional stage, retaining certain vulnerabilities of neonatal skin such as increased permeability and susceptibility to irritants, while simultaneously acquiring characteristics of adult barrier function and microbiome diversification.

As a result, infants commonly present with a unique spectrum of dermatoses distinct from those seen in the neonatal period, with a larger share attributable to environmental exposure, infections, atopic tendencies, nutrition, hygiene, and cultural practices requiring medical intervention [1]. The existing body of literature is heavily skewed toward high-volume apex institutions in urban areas, which typically see a filtered spectrum of disease and therefore obscure the true epidemiological profile in the community. A rural tertiary care center, therefore, has an inherent advantage of capturing a hybrid demography, the unreferred community infections, and witnessing the consequences of rural child-rearing practices such as oil massages, kajal application, and the early presentations of developmental anomalies before they get complicated and easily missed in the apex institutions. The current study, designed as a prospective hospital-based observational study, focused on the infantile period, excluding the neonatal period, thus excluding the physiological changes and aiming for systematic documentation of the conditions so as to guide the parents for proper skin care. A focused study identifies the local burden, modifiable risk factors, and care-seeking patterns, enabling low-cost, context-appropriate interventions, targeted training for frontline health workers, and evidence-based referral guidelines to reduce morbidity and improve infant health.

## Materials and methods

This was a hospital-based, prospective, observational study conducted in the Outpatient Department of Dermatology, Venereology, and Leprosy at Adesh Medical College and Hospital, a tertiary-care center serving a predominantly rural population in North India. The study included 200 infants aged 29 days to 12 months. The study was conducted over six months, from January 1, 2026, to June 30, 2026.

The study protocol was reviewed and approved by the Institutional Ethics Committee (IEC) and Institutional Research Committee (IRC) of Adesh Medical College and Hospital. Written informed consent was obtained from the parents or legal guardians of all enrolled infants before participation, clinical examination, photography, and publication.

Based on a reported prevalence of 36% of cutaneous manifestations among infants in a study by Bist et al. (2015) [2], the sample size was calculated using the formula:

$n = \frac{Z^{2}pq}{d^{2}}$.

With a 95% confidence level, an allowable absolute precision of 7%, and an anticipated non-response rate of 10%, the calculated sample size was approximately 200 infants. All consecutive infants aged 29 days to 12 months attending the dermatology outpatient department during the study period were included, provided that their parents or guardians gave written informed consent. Infants admitted to the pediatric intensive care unit, in whom clinical examination could compromise clinical stability, were excluded. Infants whose parents or guardians did not provide consent were also excluded.

Data were collected using a pre-designed, semi-structured proforma. Detailed demographic and clinical information was obtained, including age, sex, residence, presenting complaints, duration of lesions, relevant medical history, and history of traditional child-rearing practices. Particular attention was given to practices such as oil massage, application of kajal or cow dung, and use of home remedies.

Clinical examination was performed in a well-lit room under natural daylight whenever possible. Appropriate temperature control was maintained during examination to prevent hypothermia. Clinical photographs were obtained after informed consent from parents or guardians. Non-invasive bedside investigations, including diascopy, potassium hydroxide mount, and dermoscopy, were performed when clinically indicated. The diagnosis was made primarily on clinical grounds by a dermatologist, with bedside tests used to support the diagnosis where required.

The objective of the study was to identify, diagnose, and categorize cutaneous lesions among infants into eczematous and inflammatory, infective, congenital and developmental, and pigmentary disorders and to identify specific dermatoses that co-existed with traditional rural child-rearing practices.

Data were entered into Microsoft Excel (Microsoft Corp., Redmond, WA) and analyzed using appropriate statistical software. The data were analyzed descriptively. Categorical variables, including age group, sex, type of dermatosis, clinical diagnosis, and history of traditional child-rearing practices, were presented as frequencies and percentages.

The distribution of cutaneous lesions was described according to diagnostic categories, including eczematous and inflammatory, infective, congenital and developmental, and pigmentary dermatoses. The occurrence of specific dermatoses among infants with a history of practices such as oil massage, application of kajal or cow dung, and use of home remedies was also presented as frequencies and percentages. As no hypothesis testing or comparative statistical analysis was planned, p-values and measures of statistical significance were not calculated.

## Results

The study included 200 infants, comprising 115 males (57.5%) and 85 females (42.5%), giving a male-to-female ratio of 1.35:1 and indicating a male preponderance in the study population. Considering the age distribution, the largest proportion of infants fell in the 6-12 month age group (n = 90, 45%), followed by the 3-6 month group (n = 56, 28%) and the 29 days-3 month group (n = 52, 26%), while age data were missing/unspecified for two infants (1%).

The majority of infants belonged to a rural locality (n = 128, 64%), with the remainder from urban settings (n = 72, 36%). Based on the modified Kuppuswamy Scale [3], most families belonged to the lower-middle socioeconomic class (n = 110, 55%), followed by upper-lower (n = 47, 23.5%) and upper-middle (n = 42, 21%) classes; only one case (0.5%) belonged to the upper class (Table 1).

**Table 1:** Infant sex, age group, locality, and socioeconomic status

Characteristic	Variable/Category	Frequency (n)	Percentage (%)
Infant sex	Male	115	57.5%
	Female	85	42.5%
Infant age groups	29 days–3 months	52	26%
	3–6 months	56	28%
	6–12 months	90	45%
	Missing/Unspecified	2	1%
Locality/residence	Rural	128	64%
	Urban	72	36%
Socioeconomic status (modified Kuppuswamy)	Lower-middle	110	55%
	Upper-lower	47	23.5%
	Upper-middle	42	21%
	Upper	1	0.5%

Birth history

Regarding gestational age at birth, 129 infants (64.5%) were born at term (>37 weeks), whereas 41 (20.5%) were preterm (<37 weeks), and 30 (15%) were post-term. The mean birth weight recorded was 2.58 kg. A history of neonatal intensive care unit admission was noted in 74 infants (37%), while the remaining 126 (63%) did not require a neonatal intensive care unit stay.

Maternal history

Of the mothers, 106 (53%) were multigravida, and 94 (47%) were primigravida. Consanguinity was reported in four cases (2%), while the vast majority (98%) had no history of consanguineous marriage. A history of maternal illness during pregnancy was present in seven cases (3.5%), while 193 mothers (96.5%) had no such history.

Infant skincare practices

Daily bathing was the most common practice, reported in 139 infants (69.5%), followed by alternate-day bathing in 40 (20%) and weekly bathing in 19 (9.5%). Soap or baby soap was the predominant cleansing agent used (n = 181, 90.5%), with a minority using gram flour (besan), milk, or curd.

Oil massage was practiced in the majority of infants (n = 160, 80%), most commonly with coconut oil (n = 81, 40.5%) and baby oil (n = 55, 27.5%), followed by mustard oil (n = 21, 10.5%), desi ghee (n = 2, 1%), and olive oil (n = 1, 0.5%). Traditional cosmetic/remedial practices were also documented, with kajal/surma application being the most frequent (n = 64, 32%), followed by talcum powder use (n = 31, 15.5%), while 98 participants denied a history of application of any traditional or cosmetic product (n = 98, 49%) (Table 2).

**Table 2:** Infant skincare practices

Skincare Domain	Practice/Category	Frequency (n)	Percentage (%)
Bathing frequency	Daily	139	69.5%
	Alternate day	40	20%
	Weekly	19	9.5%
	Missing/Unspecified	2	1%
Bathing cleaning agents	Soap/Baby soap	181	90.5%
	Gram flour (Besan)	5	2.5%
	Milk	6	3%
	Curd	4	2%
	No agent used	1	0.5%
	Missing/Unspecified	3	1.5%
Oil massage practice	Yes	160	80%
	No	40	20%
Type of massage oil/fat	Coconut oil	81	40.5%
	Baby oil	55	27.5%
	Mustard oil	21	10.5%
	Ghee	2	1%
	Olive oil	1	0.5%
	Not applicable	40	20%
Traditional remedies and cosmetics	Kajal/Surma	64	32%
	Talcum powder	31	15.5%
	Cow dung	4	2%
	Honey	2	1%
	Herbal ghutti	1	0.5%
	None	98	49%

Pattern of dermatological diagnoses

A wide spectrum of dermatological conditions was observed across the study population, spanning infective (bacterial, parasitic, fungal, and viral), eczematous and inflammatory, congenital and developmental, and pigmentary categories, with few infants presenting with more than one diagnosis (Table 3).

**Table 3:** Distribution of dermatological diagnoses by category The denominator used to calculate the percentage is the total number of infants, not the total number of diagnoses, as some infants presented with multiple diagnoses, resulting in a total of 253 diagnoses.

Disease Category (n = 253)	Specific Diagnosis/Clinical Finding	Frequency (n)	Percentage of Infants (%)
Bacterial infections (n = 57)	Impetigo	48	24%
	Folliculitis	9	4.5%
Parasitic infections (n = 31)	Scabies	31	15.5%
Fungal infections (n = 33)	Candidiasis (diaper-associated)	28	14%
	Tinea	5	2.5%
Viral infections (n = 22)	Molluscum contagiosum	10	5%
	Warts	8	4%
	Hand-foot-and-mouth disease	3	1.5%
	Herpes simplex	1	0.5%
Eczematous and inflammatory (n = 93)	Atopic dermatitis	28	14%
	Diaper dermatitis	25	12.5%
	Seborrheic dermatitis/cradle cap	22	11%
	Intertrigo	2	1%
	Irritant contact dermatitis	15	7.5%
	Papular urticaria	1	0.5%
Congenital and developmental (n = 14)	Port-wine stain	1	0.5%
	Pigmented nevi	6	3%
	Hemangioma	6	3%
	Verrucous epidermal nevus	1	0.5%
Pigmentary disorders (n = 3)	Mongolian spot	2	1%
	Café-au-lait spot	1	0.5%

Depending upon the categories, eczematous and inflammatory dermatoses emerged as the single largest category overall (n = 93, 46.5%), followed by bacterial infections (n = 57, 28.5%), fungal infections (n = 33, 16.5%), scabies (n = 31, 15.5%), viral Infections (n = 22, 11%), and lastly congenital and developmental defects (n = 14, 7%) and pigmentary disorders (n = 3, 1.5%). 

The maximum number of cases of eczematous and inflammatory dermatoses reflects the high burden of conditions related to skin barrier dysfunction and irritant exposure in infancy. Atopic dermatitis, according to the Hanifin and Rajka criteria [4], was the leading diagnosis within this group (n = 28, 14%) (Figure 1), closely followed by diaper dermatitis (n = 25, 12.5%) (Figure 2), which is consistent with its well-recognized association with prolonged diaper use and seborrheic dermatitis/cradle cap (n = 22, 11%). This was followed by irritant contact dermatitis (n = 15, 7.5%) (Figure 3). Less frequent findings included intertrigo (n = 2, 1%) and papular urticaria (n = 1, 0.5%).

**Figure 1:**
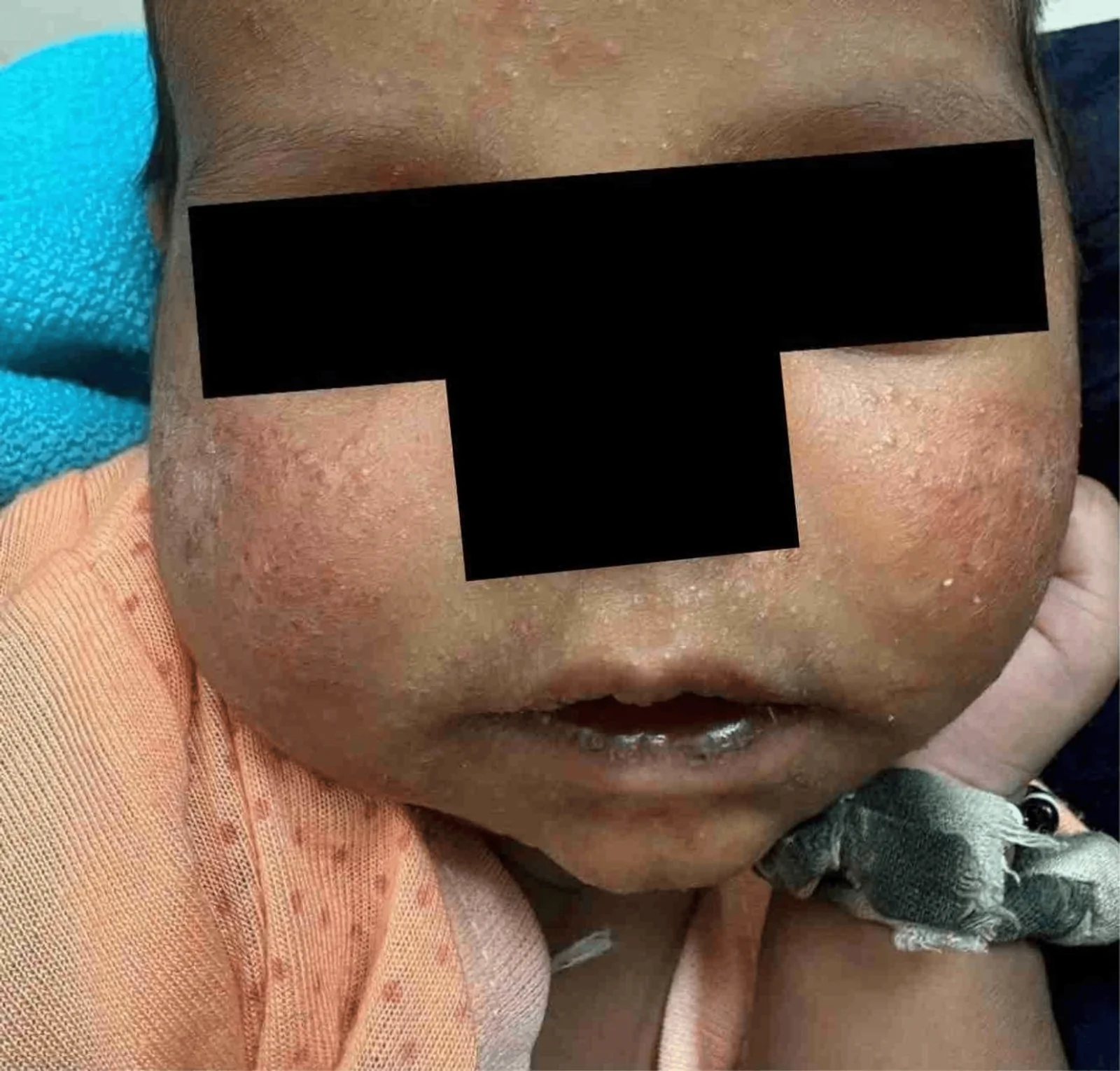
Atopic dermatitis—bilateral dry, rough, reddish to skin-colored papules on the cheeks and forehead—treated with a bland, fragrance-free moisturizer and a very thin layer of low-potency hydrocortisone ointment (1%) on the affected facial skin, twice daily for a short course, then stopped once clear

**Figure 2:**
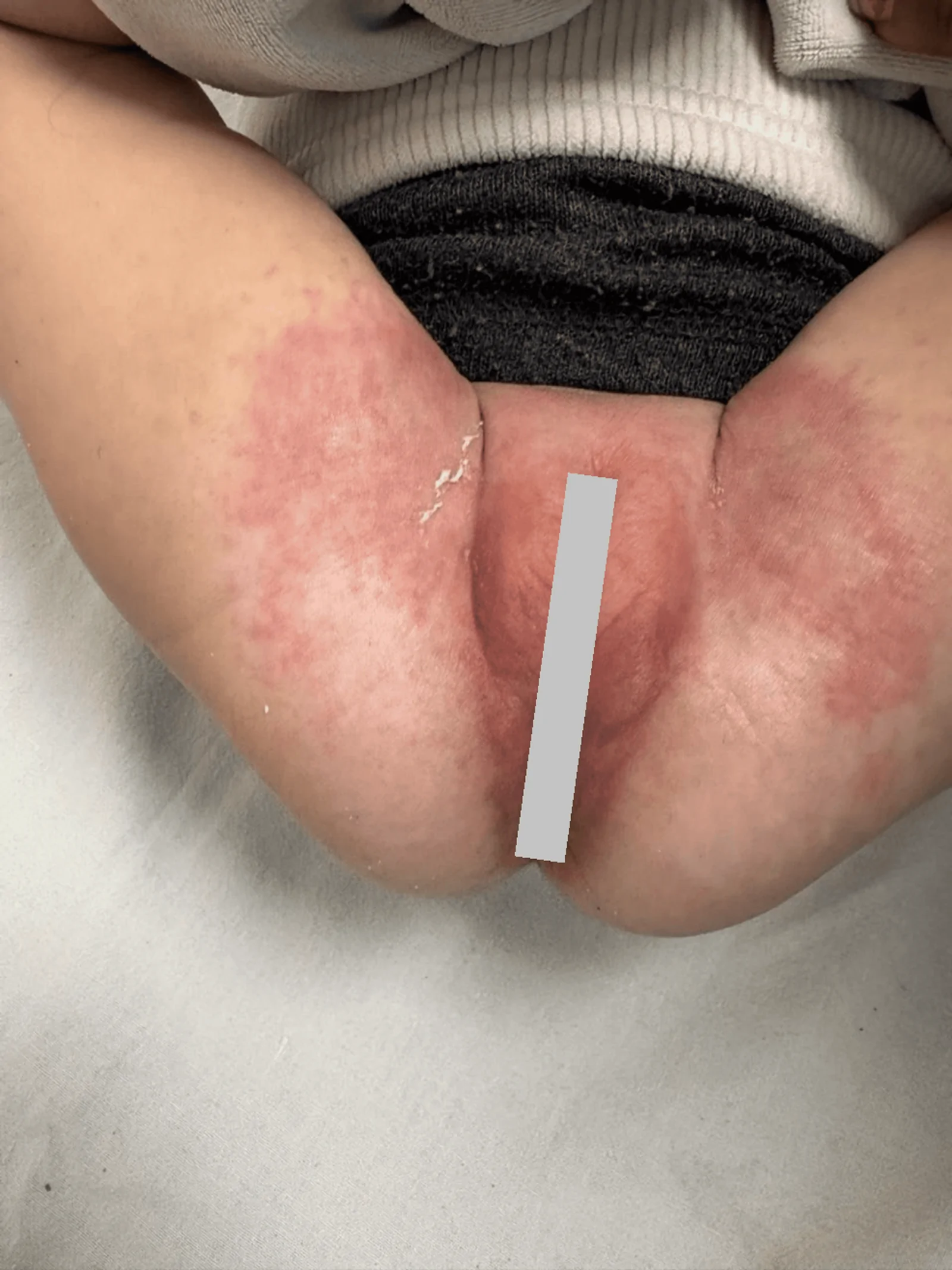
Diaper dermatitis—bilateral, fairly symmetric, erythematous rash over the genitalia, with involvement of both thighs—treated with zinc oxide barrier cream Informed parental consent was obtained for the photograph and publication.

**Figure 3:**
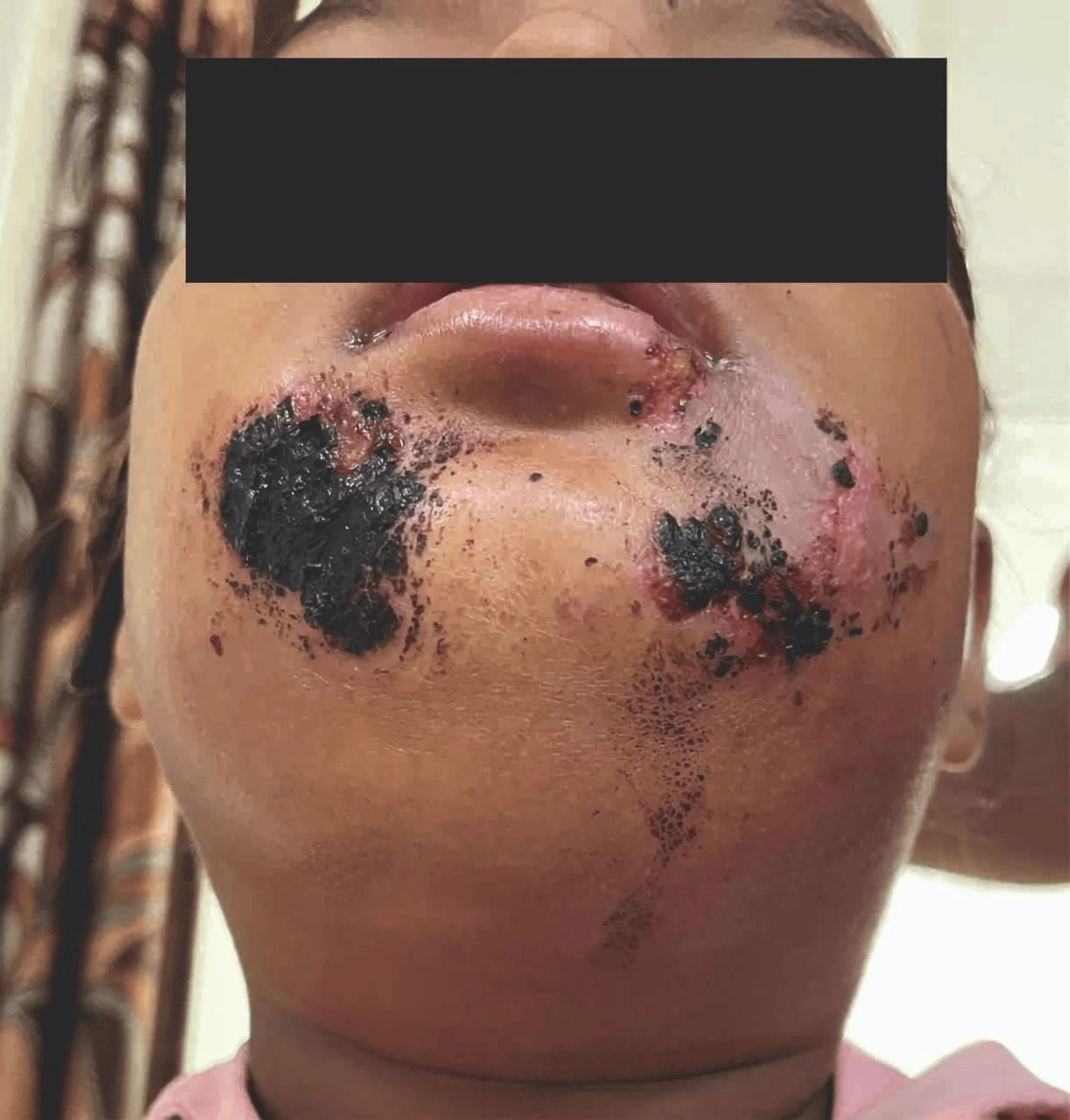
Impetigo with irritant contact dermatitis—erosive, erythematous plaques over the cheeks and chin with thick, adherent, dark crusting and facial swelling—treated with systemic antibiotics and antiallergics, along with a mild steroid-antibiotic cream in tapering doses

Bacterial infections were the next most prevalent group (n = 57, 28.5%), dominated by impetigo (n = 48, 24%), which was in fact the single most common individual diagnosis in the entire study, underscoring the vulnerability of infant skin to superficial bacterial colonization. Folliculitis accounted for a smaller share (n = 9, 4.5%). Fungal infections were noted in 33 cases (n = 33, 16.5%), with candidiasis as the majority (n = 28, 14%) (Figure 4), while superficial dermatophytic tinea infections were comparatively uncommon (n = 5, 2.5%).

**Figure 4:**
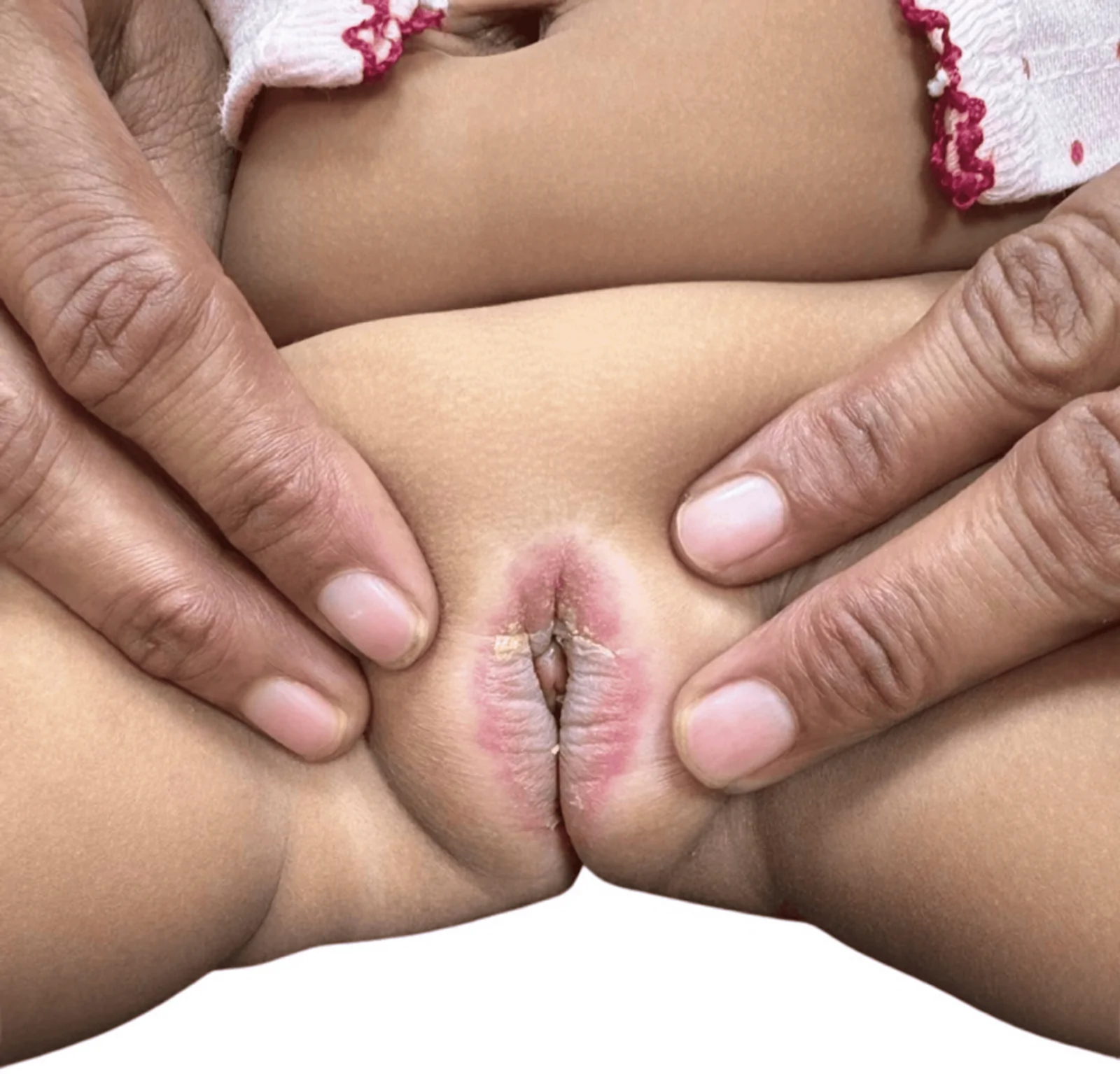
Candidiasis—erythematous, moist, sharply demarcated rash—treated with topical antifungal cream Informed parental consent was obtained for the photograph and publication.

Parasitic infestation, represented solely by scabies, was seen in 31 cases (n = 31, 15.5%), making it individually the second most common diagnosis after impetigo and pointing to a significant burden of infestation-related dermatoses, likely reflecting household and hygiene-related transmission patterns. Viral infections together accounted for 22 cases (n = 22, 11%), led by molluscum contagiosum (n = 10, 5%) and viral warts (n = 8, 4%), with hand-foot-and-mouth disease (n = 3, 1.5%) (Figure 5) and herpes simplex (n = 1, 0.5%) forming a minor share.

**Figure 5:**
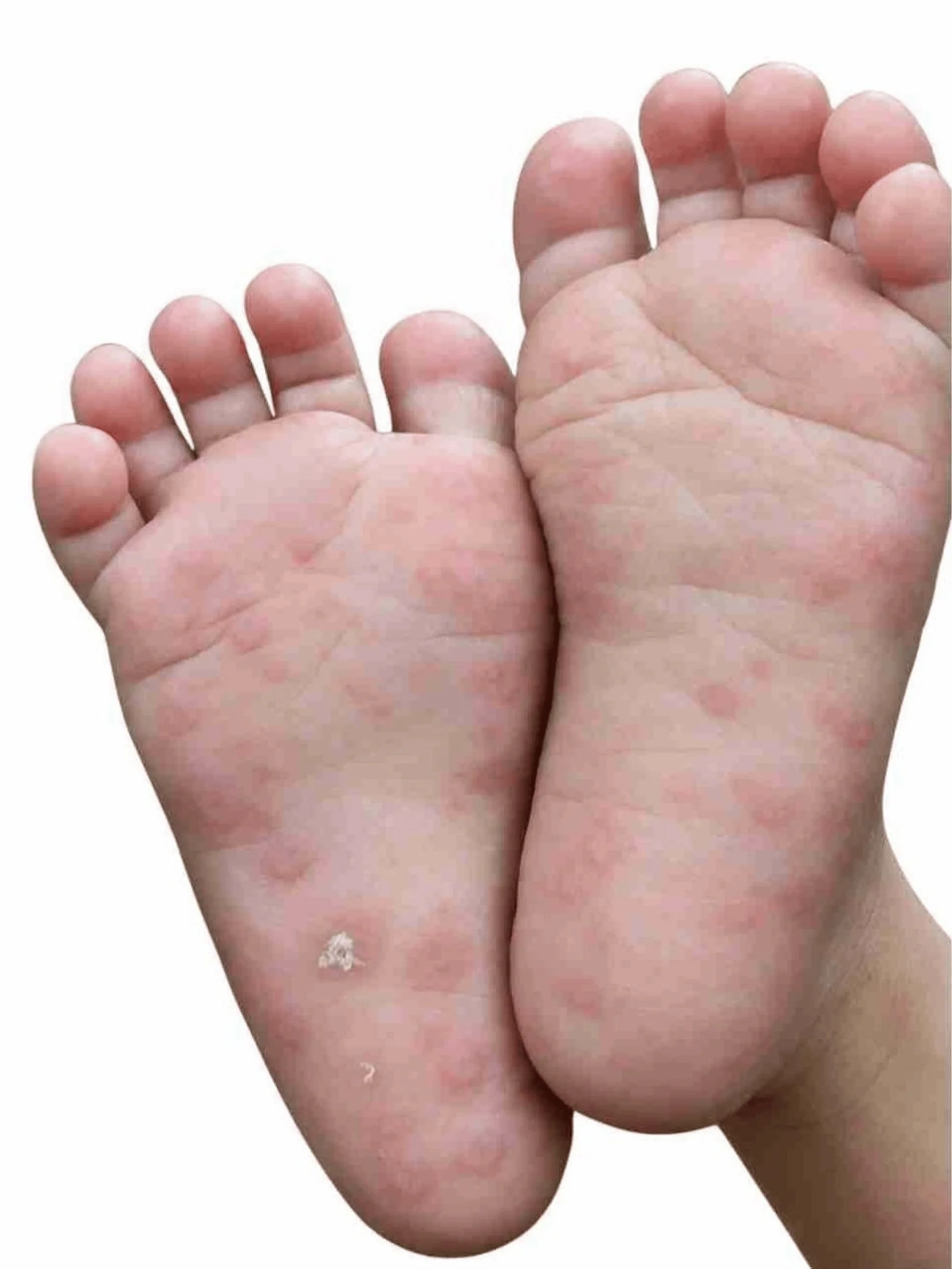
Hand-foot-and-mouth disease—scattered, symmetric red macules and papules on both soles, with some lesions progressing to small blisters—treated symptomatically, and the lesions resolved within two weeks

Congenital and developmental lesions were identified in 14 infants (n = 14, 7%), the most frequent being pigmented nevi (n = 6, 3%) and hemangioma (n = 6, 3%) (Figure 6). Rarer findings included port-wine stain and verrucous epidermal nevus (n = 1, 0.5% each) (Figure 7).

**Figure 6:**
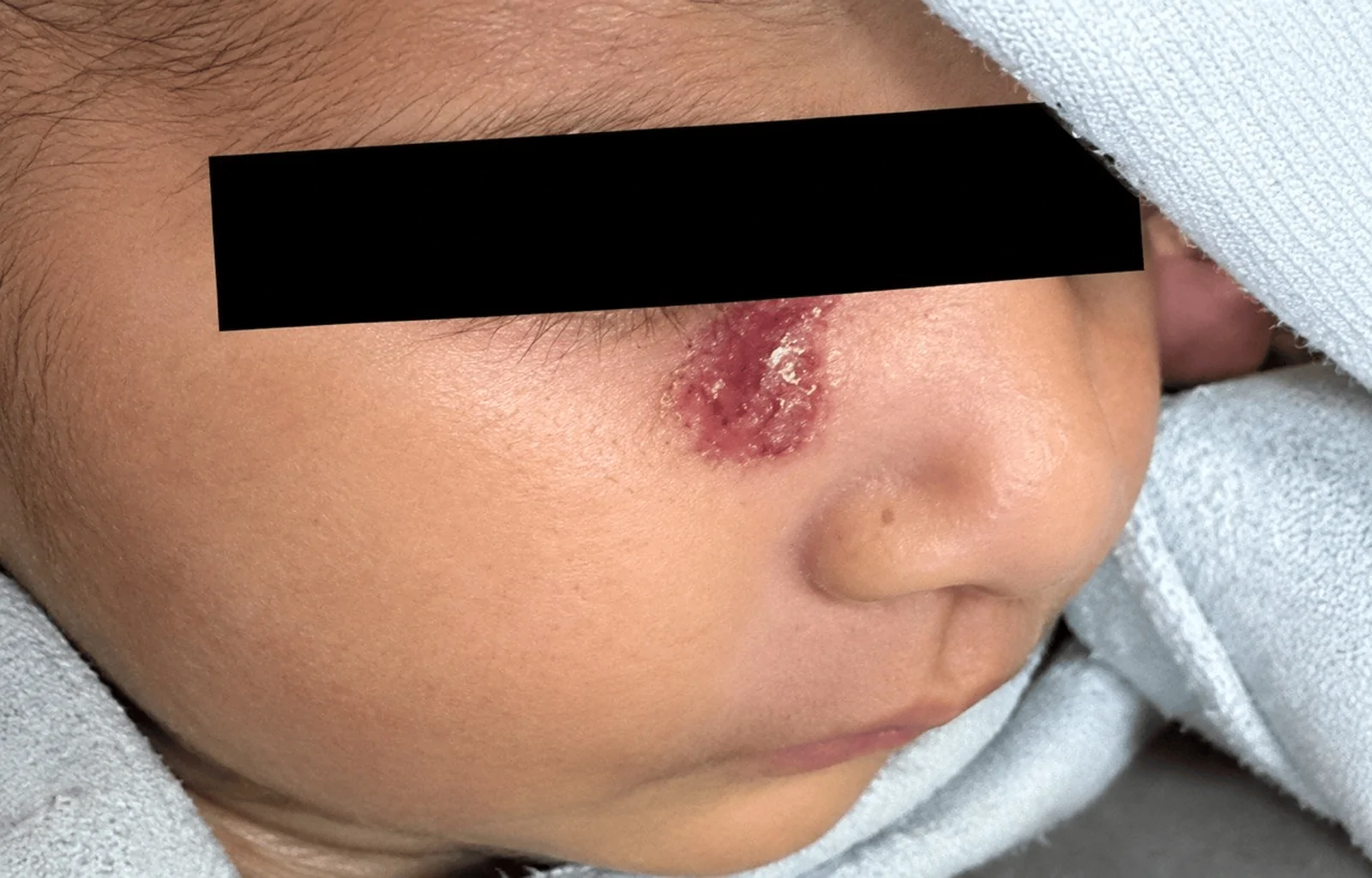
Infantile hemangioma—a raised, well-defined, red-purple vascular plaque on the medial side of the cheek—treated with one drop of 0.5% timolol gel twice daily, with regular follow-ups every two to three months for dose adjustment and monitoring

**Figure 7:**
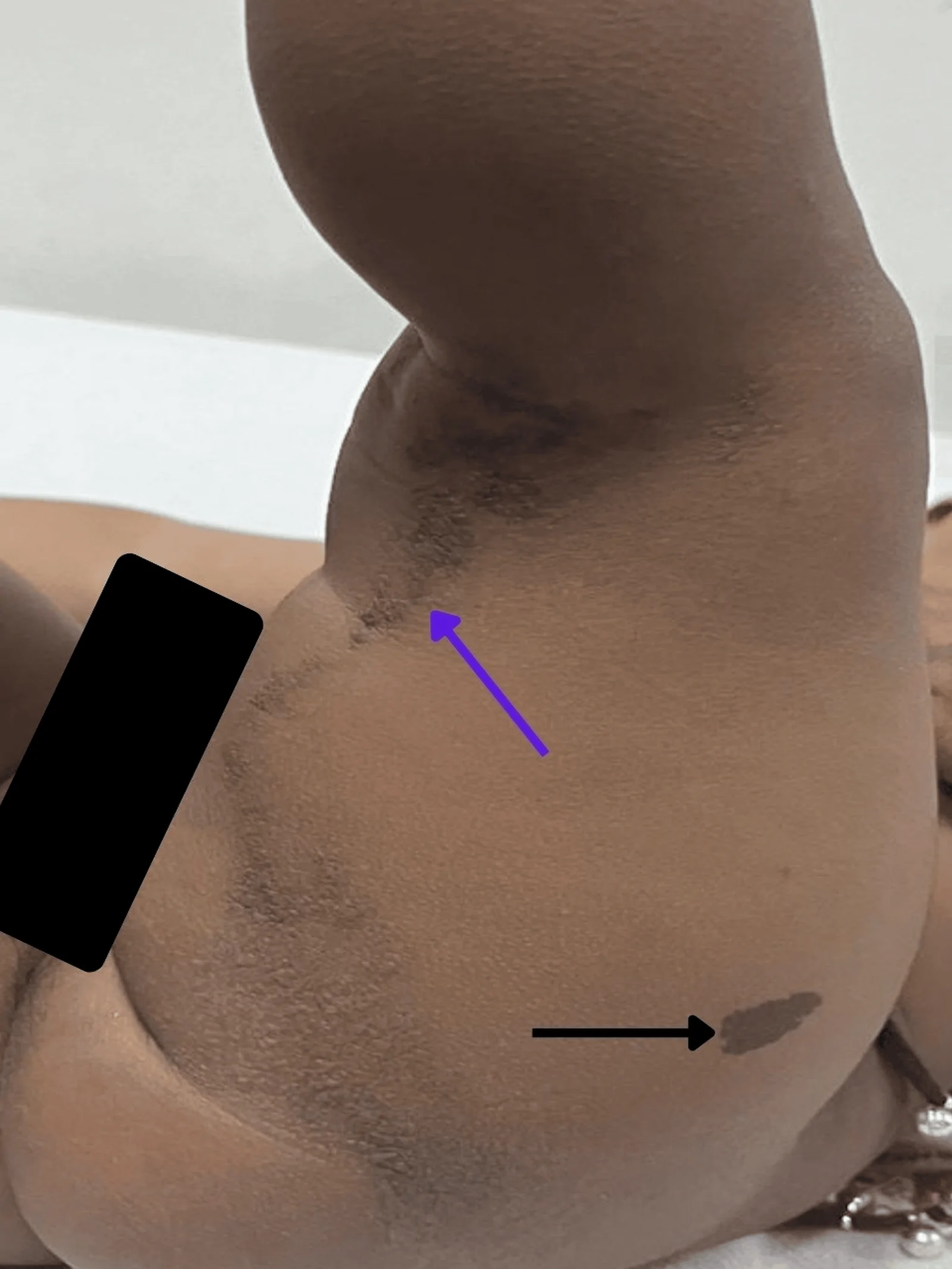
Verrucous epidermal nevus (purple arrow)—well-demarcated, hyperpigmented to skin-colored, verrucous, and hyperkeratotic papules and thin plaques following the lines of Blaschko present over the posterior aspect of the left thigh, along with a café-au-lait spot (black arrow)—a solitary, well-defined, uniformly pigmented, brown-colored macule with a smooth surface and no induration or scaling. The parents were counseled about the benign nature of the lesions, and the patient was prescribed bland emollients and advised annual follow-ups.

Pigmentary disorders were the least represented category, with Mongolian spot (Figure 8) recorded in only two cases (n = 2, 1%) and cafe-au-lait spot in one case (n = 1, 0.5%) (Figure 7).

**Figure 8:**
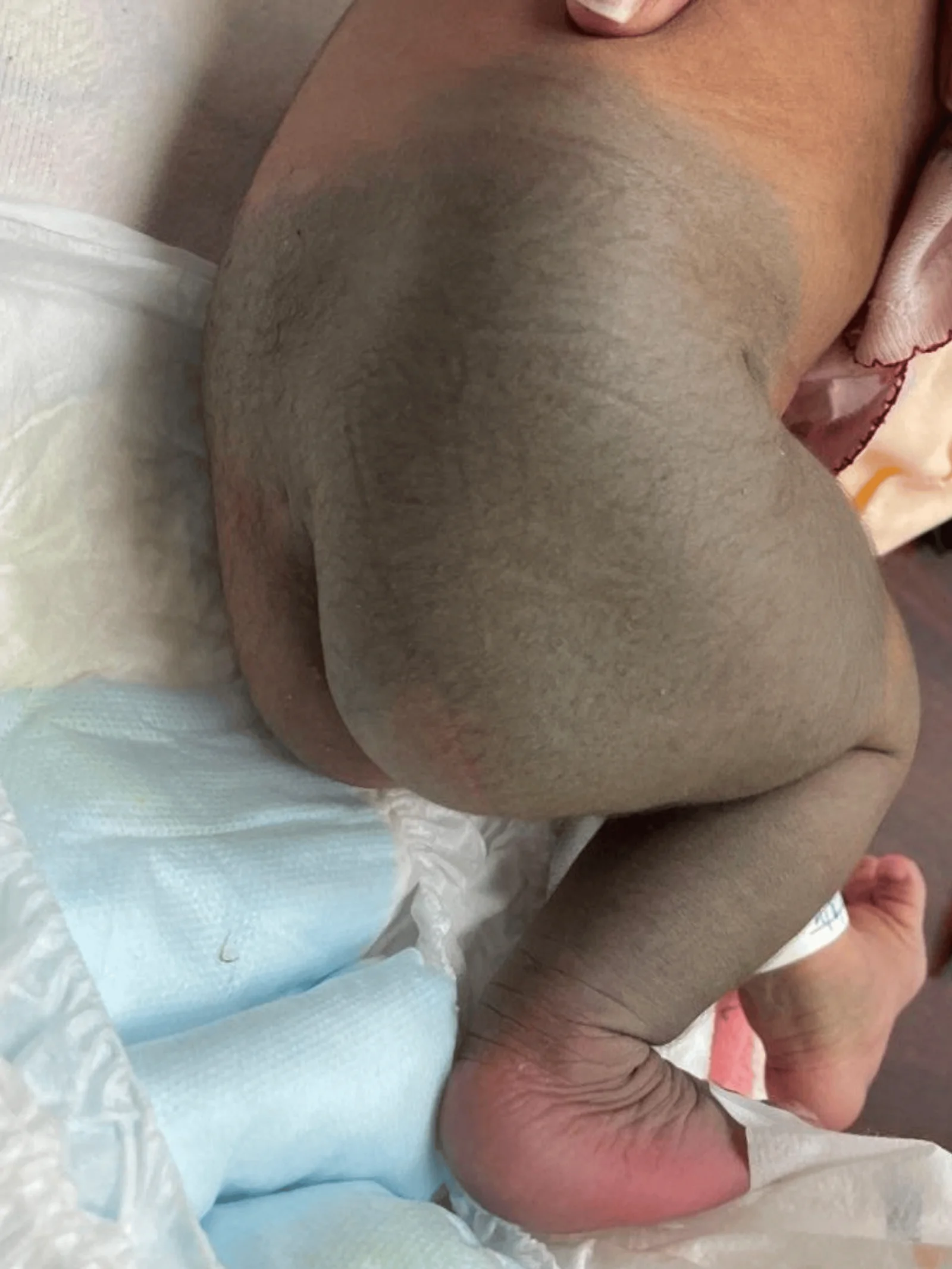
Mongolian spot—a congenital, flat, slate-blue-colored, large patch with irregular but well-defined margins covering the whole of the sacrococcygeal region and extending to the bilateral lower limbs. The parents were counseled and reassured about the benign nature of the lesion and advised annual follow-ups.

Overall, this pattern highlights that eczematous/inflammatory conditions (atopic dermatitis, diaper dermatitis, seborrheic dermatitis, irritant contact dermatitis, and intertrigo) and infective dermatoses (bacterial, parasitic, fungal, and viral infections combined) together accounted for the vast majority of presentations, with congenital and pigmentary lesions forming a comparatively minor but clinically important subset.

## Discussion

Extensive research has been conducted to assess the cutaneous manifestations in infants, although the spectrum of disease varies significantly based on geography, climatic conditions, and socioeconomic status. Ethnicity also plays a role. There are many studies on infants, with a special focus on neonates. The cutaneous findings in neonates include physiological changes along with pathological changes. The present study examined 200 infants aged 29 days to 12 months presenting to the dermatology outpatient department of a tertiary center serving a predominantly rural catchment. Its defining structural feature is the age window itself. By beginning at about one month, the series excludes almost the entire physiological repertoire that dominates newborn surveys, and the resulting spectrum differs accordingly. Some cutaneous findings may persist in an infant well beyond the neonatal period.

Eczematous and inflammatory dermatoses formed the largest single category, with 93 diagnoses (46.5% of infants). Atopic dermatitis, according to the Hanifin and Rajka criteria, was the leading diagnosis, with 28 cases (14%), followed by diaper dermatitis (n = 25, 12.5%) and seborrheic dermatitis or cradle cap (n = 22, 11%). Seborrheic dermatitis was the most common eczema among Uttarakhand infants, where atopic dermatitis accounted for 19.04% of non-infectious dermatitis or 6.84% of infants [2]. The internal ordering departs from the comparison series: dermatitis in the Delhi cohort was led by infantile seborrheic dermatitis and cradle cap [5]. Two explanations are available within these data, and neither can be separated from the other here. The first is age structure: the largest age stratum in the present series was six to 12 months (n = 90, 45%), whereas seborrheic dermatitis is characteristically an earlier-infancy diagnosis, and atopic dermatitis presents in 45% of cases within the first six months and 60% within the first year [6]. The second is the diagnostic threshold. Atopic dermatitis is a clinical diagnosis requiring pruritus plus at least three of the typical features, including the morphology and distribution of lesions, chronicity, and personal or family history of atopy, along with more than three minor criteria according to Hanifin and Rajka, or pruritus along with a personal or first-degree family history of atopy, dry skin, a history or presence of flexural dermatitis, and onset before two years according to the UK Working Party diagnostic criteria [7], the latter carrying an explicit caveat below the age of four [6]. In infancy, the morphology is papulovesicular, with exudate or crusting on the cheeks, scalp, and neck in an extensor rather than flexural distribution, which overlaps materially with both seborrheic dermatitis and infantile scabies [2,5,6,8,9]. Without follow-up, a cross-sectional label of atopic dermatitis at this age is necessarily provisional, and the 14% figure is best presented as such. Sardana et al. (2009) analyzed the pediatric dermatological spectrum in Delhi and reported a high prevalence of diaper dermatitis (18%), associating it with the use of occlusive cloth napkins and infrequent changing practices common in resource-limited settings [5]. Timilsina et al. (2024) in Nepal observed that while physiological lesions constituted the bulk of findings, infectious dermatoses (cradle cap, napkin dermatitis, and impetigo) accounted for 5.31% of cases. This incidence is notably higher than Western figures, likely due to environmental factors [10].

In the present study, infective diseases made the dominant contribution. Bacterial, parasitic, fungal, and viral diagnoses together accounted for 143 of the 253 recorded diagnoses (56.5% of the total diagnoses), and impetigo alone, at 48 cases (24% of the study population), was the single most common diagnosis in the series. This reproduces the hierarchy established in the largest available Indian pediatric dataset, in which infections and infestations constituted 47.15% of 32,341 new dermatoses among children under 12 years, with bacterial pyodermas forming 58.09% of that group [5]. Thappa's review of the Indian pediatric outpatient burden identified the same case mix of pyoderma, molluscum, warts, dermatophytes, and candidiasis [11]. Bist et al. found infectious or parasitic disease in 26.92% of 234 infants at an Uttarakhand tertiary facility [2]. The bacterial share in our study (57 of 143 infective diagnoses, 39.9%) is lower than the share reported by Sardana et al. (58.09%) [5], which is consistent with a narrower age band and a clinic rather than a whole-childhood referral spectrum. In a study by Nanda et al., impetigo neonatorum reached 11.3% in Chandigarh during the hot, humid months of May to August [12], and nonfungal, nonparasitic infections rose in summer in the Uttarakhand infant series [2]; the present recruitment period of January to June straddles winter and early summer but excludes the monsoon, so the 24% figure should be read as a seasonally partial estimate rather than an annual one. That guidance rests on well-established structural grounds - a higher surface-area-to-weight ratio, weaker intercellular attachments, and greater percutaneous absorption than adult skin [13-15].

Fungal infections (n = 33, 16.5%) included candidiasis (14%) as well as dermatophytic tinea infection (2.5%). The higher number of fungal infections observed in our study may be associated with the predominantly rural background of the study population. Environmental factors, including increased exposure to humid conditions, together with limited awareness regarding hygiene, early recognition of infection, and timely treatment, may predispose to fungal infections. In addition, childcare practices such as inadequate drying of flexural areas and prolonged exposure to moisture may facilitate fungal overgrowth. Conversely, inappropriate use of topical preparations, particularly potentially irritant or systemically absorbable agents, may further alter the cutaneous barrier and contribute to susceptibility to infection [15].

Among parasitic infections, scabies, at 31 cases (15.5%), was the second most common individual diagnosis after impetigo and the only parasitic infestation recorded. Expressed against infective diagnoses, it constituted 21.7%, closely matching the 21.54% share of scabies within infections and infestations reported from Delhi [5]. It exceeds the 7.26% recorded among Uttarakhand infants [2] and the 6.1% prevalence among children at a peripheral Kashmiri health center, where the mean age was 2.4 years and cases clustered in colder months and in rural, lower-income households. Both the rural preponderance (64%) and the lower-middle and upper-lower socioeconomic distribution (78.5% combined) of the present cohort align with that transmission profile [9]. Scabies in infancy is not the disease of the adult; burrows may be absent, bullae and pustules are relatively common, and the scalp, neck, and face may be involved [8,9]. Thappa (2002) reinforced this, noting that in rural areas, untreated infantile scabies often serves as the reservoir for household reinfestation [11]. Bhat et al. (2017) conducted a community-based study in rural Kashmir and identified scabies as a major health burden, affecting 12.8% of the population. They emphasized that scabies often presents atypically (involving the palms, soles, and face), leading to misdiagnosis by peripheral health workers [16].

Congenital and developmental lesions included pigmented nevi (n = 6, 3%), hemangioma (n = 6, 3%), port-wine stain, and verrucous epidermal nevus (n = 1, 0.5% each), while pigmentary lesions included Mongolian spot (n = 2, 1%) and café-au-lait spot (n = 1, 0.5%). In a study by Moosavi and Hosseini (2006) in Iran, 96% of infants exhibited cutaneous lesions, among which the most prevalent were Mongolian spots (71.3%) [17]. Similar findings were seen in a study by Ferahbas et al. (2009) in Turkey, which emphasized that ethnicity dictates the frequency of pigmentary lesions such as Mongolian spots (67.3%), which are ubiquitous in Asians but rare in Caucasians [18]. Nanda et al. (1989) conducted a landmark survey in North India and reported Mongolian spots in 89% of the study population [12]. Dash et al. (2000) in Odisha corroborated these findings, noting Mongolian spots (60%) and milia (31%) as predominant findings. Their study highlighted that differentiation of these benign lesions from pathological ones is critical to preventing parental anxiety [19]. While physiological lesions dominate the landscape, pathological conditions contribute significantly to morbidity, especially in developing nations.

In the Indian subcontinent, various child-rearing practices involving infant skin care are followed and are influenced by cultural diversity. Various traditional and customary child-rearing practices were observed in the study population. Kajal application was the most frequently reported practice, noted in 64 infants (n = 64, 32%), followed by talcum powder (n = 31, 15.5%), cow dung (n = 4, 2%), honey (n = 2, 1%), and herbal ghutti (n = 1, 0.5%). Irritant contact dermatitis was observed in 15 cases. Among these, 10 cases were seen in infants with talcum powder application, three with cow dung application, and two with kajal application. Surprisingly, kajal application was the most frequently reported practice, but only two cases reported a reaction to it. Talcum powder application was the most commonly reported child-rearing practice among infants who developed irritant contact dermatitis, followed by cow dung and kajal application. These observations suggest the potential role of traditional and commonly used childcare practices in contributing to irritant skin reactions in infants. Baruah et al. (1991) in Pondicherry noted specific dermatoses related to the application of kajal and talcum powder, which are pervasive practices in rural households [20], similar to the findings in our study. Dhar (2007) revisited infant skin care, highlighting that traditional oil massages (using mustard oil or ghee) can have variable effects; while some oils improve barrier function, others may induce irritant contact dermatitis or folliculitis [15]. This is in contrast to our study, as none of the cases reported any reactions to the application of oil. In a study by Karampinis et al., the dermoscopic presentations of pediatric skin diseases in skin of color often differ from those in Caucasian populations due to increased melanin levels that alter backgrounds and obscure classic vascular signs. A literature review of 61 studies indicated that while certain conditions exhibit darker backgrounds or race-specific markers, other dermatological features remain uniform across all skin types [21].

Limitations

The present study has certain limitations that should be considered while interpreting its findings. First, this was a single-center study conducted in a specific healthcare setting. There may be selection/referral bias, as it is a specific departmental study. Therefore, the results may not be fully generalizable to other regions, institutions, or populations with differing geographic, cultural, socioeconomic, and healthcare-delivery characteristics. Multicenter studies involving diverse settings would help establish whether the observed trends are consistent across broader populations. Second, the study population predominantly comprised individuals from a rural background. The pattern and frequency of dermatoses identified in this study may consequently reflect local demographic characteristics, socioeconomic conditions, occupational and environmental exposures, hygiene practices, health awareness, and healthcare-seeking behavior. These factors may differ substantially in urban or semi-urban populations, potentially resulting in a different spectrum of dermatological conditions. Finally, the study was conducted over a relatively short period of six months. This duration may have limited the ability to assess seasonal variations in the occurrence and distribution of dermatoses, particularly conditions influenced by climatic factors such as temperature, humidity, rainfall, and seasonal exposure patterns. A longer prospective study spanning an entire year or multiple years would be better suited to capturing these variations and providing a more comprehensive understanding of the epidemiological pattern of dermatoses.

## Conclusions

Most published studies on infantile dermatoses have been conducted at high-volume apex or referral institutes, with relatively limited literature from lower-volume tertiary care centers that serve as the first point of referral for predominantly rural populations. Such centers provide an important surveillance point for understanding the spectrum and distribution of dermatological disorders in the community, particularly in populations with limited access to specialized dermatological care. Several studies have documented the spectrum of dermatoses occurring during infancy, with many focusing predominantly on the neonatal period. In contrast, our study specifically evaluates infants aged 29 days to 12 months, thereby excluding the neonatal period. This approach minimizes the representation of physiological neonatal conditions, congenital anomalies, and other dermatoses predominantly encountered during the neonatal period. Instead, the study focuses primarily on acquired infectious and inflammatory dermatoses, which constitute an important proportion of dermatological disorders encountered during the post-neonatal period. Infants in this age group may present with delayed or complicated disease, including mixed infections and secondary complications, partly due to the use of home remedies or delayed healthcare-seeking. Conversely, some dermatological lesions may be detected incidentally during routine immunization visits, providing an opportunity for early recognition and intervention. A focused evaluation of dermatoses in this relatively less-studied age group can therefore provide valuable insights into disease distribution, preventable dermatological morbidity, and potentially modifiable household practices. The findings may also help identify areas for improved parental education, early referral, and preventive dermatological care among infants from predominantly rural populations.

## References

[1] Burns T, Breathnach S, Cox N, Griffiths C (2010). Rook's Textbook of Dermatology. Rook's Textbook of Dermatology. 8th ed. Chichester, West Sussex: Wiley-Blackwell.

[2] Bist JS, Rana SK, Kumari N, Singh R, Mehta AK (2015). Prevalence of skin diseases among infants in a tertiary medical facility in Uttarakhand. Int J Res Med Sci.

[3] Katre R, Singh P, Grover K (2026). Modified Kuppuswamy scale 2026: methodological comparison of approaches for updating income classification. Int J Community Med Public Health.

[4] Hanifin JM, Rajka G (1980). Diagnostic features of atopic dermatitis. Acta Derm Venereol.

[5] Sardana K, Mahajan S, Sarkar R, Mendiratta V, Bhushan P, Koranne RV, Garg VK (2009). The spectrum of skin disease among Indian children. Pediatr Dermatol.

[6] Ahn C, Huang W (2024). Clinical presentation of atopic dermatitis. Adv Exp Med Biol.

[7] Williams HC, Burney PG, Hay RJ (1994). The U.K. Working Party's Diagnostic Criteria for Atopic Dermatitis. I. Derivation of a minimum set of discriminators for atopic dermatitis. Br J Dermatol.

[8] Chiriac A, Diaconeasa A, Miulescu R, Chiriac AE, Wollina U (2024). Scabies in infants and children - a narrative review. Eur J Pediatr.

[9] Pir IA, Gani B, ul Islam J (2021). Clinico-epidemiological profile of scabies among children in a peripheral health centre in Kashmir, India. Int J Recent Adv Multidiscip Topics.

[10] Timilsina A, Bhatta NK, Shah N (2024). Clinical study of cutaneous manifestations in neonates in a tertiary care center in Eastern Nepal: a descriptive cross-sectional study. JNMA J Nepal Med Assoc.

[11] Thappa DM (2002). Common skin problems. Indian J Pediatr.

[12] Nanda A, Kaur S, Bhakoo ON, Dhall K (1989). Survey of cutaneous lesions in Indian newborns. Pediatr Dermatol.

[13] Usmani SZ, Saxena K, Koti V, Bhatnagar S, Paliwal G, Yadav S, Singh A (2019). A clinical study of various cutaneous manifestations in neonates. Int J Res Dermatol.

[14] Sneha M, Sadagopan K, Vaishnavi D (2021). A clinical study of cutaneous manifestations in neonates at a tertiary health care center. Int J Res Dermatol.

[15] Dhar S (2007). Newborn skin care revisited. Indian J Dermatol.

[16] Bhat SA, Mounsey KE, Liu X, Walton SF (2017). Host immune responses to the itch mite, Sarcoptes scabiei, in humans. Parasit Vectors.

[17] Moosavi Z, Hosseini T (2006). One-year survey of cutaneous lesions in 1000 consecutive Iranian newborns. Pediatr Dermatol.

[18] Ferahbas A, Utas S, Akcakus M, Gunes T, Mistik S (2009). Prevalence of cutaneous findings in hospitalized neonates: a prospective observational study. Pediatr Dermatol.

[19] Dash K, Grover S, Radhakrishnan S, Vani M (2000). Clinico epidemiological study of cutaneous manifestations in the neonate. Indian J Dermatol Venereol Leprol.

[20] Baruah MC, Bhat V, Bhargava R, Garg RB, Kumar V (1991). Prevalence of dermatoses in the neonates in Pondicherry. Indian J Dermatol Venereol Leprol.

[21] Karampinis E, Toli O, Georgopoulou KE (2024). Exploring pediatric dermatology in skin of color: focus on dermoscopy. Life (Basel).

